# Ethosomes for Coenzyme Q10 Cutaneous Administration: From Design to 3D Skin Tissue Evaluation

**DOI:** 10.3390/antiox9060485

**Published:** 2020-06-03

**Authors:** Maddalena Sguizzato, Paolo Mariani, Francesco Spinozzi, Mascia Benedusi, Franco Cervellati, Rita Cortesi, Markus Drechsler, Roxane Prieux, Giuseppe Valacchi, Elisabetta Esposito

**Affiliations:** 1 Department of Chemical and Pharmaceutical Sciences, University of Ferrara, I-44121 Ferrara, Italy; sgzmdl@unife.it; 2 Department of Life and Environmental Sciences, Polytechnic University of Marche, I-60131 Ancona, Italy; p.mariani@staff.univpm.it (P.M.); f.spinozzi@univpm.it (F.S.); 3 Department of Biomedical and Specialist Surgical Sciences, University of Ferrara, I-44121 Ferrara, Italy; mascia.benedusi@unife.it (M.B.); franco.cervellati@unife.it (F.C.); prxrnf@unife.it (R.P.); 4 Bavarian Polymer Institute (BPI) Keylab “Electron and Optical Microscopy”, University of Bayreuth, D-95440 Bayreuth, Germany; Markus.Drechsler@uni-bayreuth.de; 5 Animal Science Dept., Plants for Human Health Institute, NC Research Campus, NC State University, Kannapolis, NC 28081, USA; 6 Department of Food and Nutrition, Kyung Hee University, Hoegi-Dong, Dongdaemun-Gu, Seoul 130-701, Korea

**Keywords:** ethosome, ubiquinone, H_2_O_2_, penetration enhancers, dermal administration, reconstituted human epidermis, small angle X-ray scattering

## Abstract

Ethosome represents a smart transdermal vehicle suitable for solubilization and cutaneous application of drugs. Coenzyme Q10 is an endogenous antioxidant whose supplementation can counteract many cutaneous disorders and pathologies. In this respect, the present study describes the production, characterization, and cutaneous protection of phosphatidylcholine based ethosomes as percutaneous delivery systems for coenzyme Q10. CoQ10 entrapment capacity in ethosomes was almost 100%, vesicles showed the typical ‘fingerprint’ structure, while mean diameters were around 270 nm, undergoing an 8% increase after 3 months from production. An ex-vivo study, conducted by transmission electron microscopy, could detect the uptake of ethosomes in human skin fibroblasts and the passage of the vesicles through 3D reconstituted human epidermis. Immunofluorescence analyses were carried on both on fibroblasts and 3D reconstituted human epidermis treated with ethosomes in the presence of H_2_O_2_ as oxidative stress challenger, evaluating 4-hydroxynonenal protein adducts which is as a reliable biomarker for oxidative damage. Notably, the pretreatment with CoQ10 loaded in ethosomes exerted a consistent protective effect against oxidative stress, in both models, fibroblasts and in reconstituted human epidermis respectively.

## 1. Introduction

Coenzyme Q10 (CoQ10) is a lipophilic endogenous antioxidant involved in the production of cellular energy, having a key role in the mitochondria electron transport chain [1,2]. Particularly, during the aerobic cellular respiration, CoQ10 gives rise to adenosine triphosphate, thus producing cellular energy. In the meantime CoQ10 is reduced to ubiquinol, the form able to prevent cell membrane peroxidation [3]. Since CoQ10 tissue level decreases with aging, its supplementation is indicated in many geriatric disorders and pathologies, for instance hearing loss, diabetes, Parkinson’s and cardiovascular diseases [4,5]. Notably, CoQ10 can be employed as adjuvant therapy in cancer patients, due to its immunostimulatory ability [6,7,8]. Many studies have demonstrated that the supplementation of CoQ10 can improve the antioxidant properties of skin, decreasing reactive oxygen species and thus leading to long-term anti-aging effects [9,10,11]. Moreover, it has been demonstrated that the cutaneous administration of CoQ10 is suitable for skin photoprotection [12,13,14,15]. Notwithstanding its beneficial role, CoQ10 administration through the skin can be problematic, due to the low drug solubility. In this regard some authors have proposed the nanoencapsulation of CoQ10 within different devices able to solubilize and deliver CoQ10 onto the skin [14,15,16,17,18,19,20]. For instance, polymeric or solid lipid nanoparticles, nanostructured lipid carriers, and liposomes have been investigated, suggesting their suitability for CoQ10 topical delivery. Nonetheless, to the best of our knowledge, the possibility to entrap CoQ10 within ethosomes (ETHO) has never been described. ETHO are vesicular systems whose composition is mainly based on phospholipids (e.g., phosphatidylcholine, PC), ethanol, and water [21,22]. ETHO can be defined as the second generation of liposome, in which the relatively high percentage of ethanol (20–45%) makes the vesicles more stable and enables them to achieve higher drug entrapment efficiency with respect to classic liposomes [21,22,23,24]. Notably, in vitro and in vivo studies have demonstrated that ETHO possess a transdermal potential, making them suitable for enhancing absorption and bioavailability of different drugs [25,26,27,28,29]. The transdermal effect has been attributed to the synergistic properties of PC and ethanol, acting both as penetration enhancers. Indeed, on one hand ethanol makes the vesicle structure softer than conventional liposomes, on the other PC and ethanol exert a penetration enhancement effect [23,24,25,26,27]. Particularly, PC itself can promote skin permeation of actives in reason of its chemical similarity with skin lipids [30], while ethanol opens spaces within the stratum corneum, facilitating the delivery of therapeutic and cosmetic agents through the skin [31]. On this matter it is supposed that, due to their softness, ETHO can pass intact through the modified stratum corneum, while they fuse with the lipids in the deeper skin layers [21,22]. In this scenario, the possibility to achieve CoQ10 dermal/transdermal delivery by ETHO has appeared particularly appealing. Namely, the first part of the present study describes the design and characterization of CoQ10 containing ETHO, while the second part deals with an ex-vivo study aimed at investigating the behavior of CoQ10 loaded in ETHO applied on the skin. Particularly, the uptake of ETHO in primary human skin fibroblasts has been evaluated by transmission electron microscopy (TEM), while the antioxidative effect of CoQ10 has been studied by immunocytochemical analysis, evaluating the biomarker for oxidative stress 4-hydroxynonenal (4-HNE) on cells treated with H_2_O_2_ as oxidative stress challenger. Finally, the inhibition of the oxidative damage induced by H_2_O_2_ has been evaluated also on reconstituted human epidermis (RHE) treated with CoQ10 loaded ETHO.

## 2. Materials and Methods

### 2.1. Materials

Coenzyme Q10 (ubiquinone, CoQ10) was purchased from Sigma-Aldrich (St Louis, MO, USA). The soybean lecithin (PC) (90% phosphatidylcholine) used for ETHO preparation was Epikuron 200 from Lucas Meyer, Hamburg, Germany. Solvents were HPLC grade and all other chemicals were analytical grade.

### 2.2. CoQ10 Solubility Evaluation

Solubility of CoQ10 in water, ethanol, methanol, dimethylsufoxide, and acetonitrile was determined by saturating each solvent or solvent mixture with an excess of drug. The obtained saturated solutions were mixed in an horizontal shaker (100 rpm) in the dark for 3 h. Afterwards 1 mL of samples were withdrawn and filtered through a Millex-LCR Filter, 0.45 μm, hydrophilic PTFE, 25 mm (Millipore-Sigma-Aldrich Merck, Darmstadt, Germany). CoQ10 concentration was determined by reverse phase high performance liquid chromatography (RP-HPLC) analysis. HPLC analyses have been conducted using a quaternary pump (Agilent Technologies 1200 series, Santa Clara, CA, USA), a UV-detector operating at 275 nm, and a 7125 Rheodyne injection valve with a 50 μL loop. Samples have been loaded on a stainless steel C-18 reverse-phase column (15 × 0.46 cm) packed with 5 μm particles (Grace^®^ - Alltima, Alltech, Bareggio, Milano, Italy). A mobile phase constituted of acetonitrile:tetrahydrofuran:water (55:40:5, *v*/*v*/*v*) has been eluted at a 1 mL/min flow rate, retention time was 5.7 min. The method was validated for linearity (*R*^2^ = 0.995), repeatability (relative standard deviation 0.02%, *n* = 6 injections), and limit of quantification (0.04 µg/mL). Analyses were conducted in triplicate, mean and standard deviations values were calculated.

### 2.3. Production of Ethosomes

For the preparation of ETHO, PC (15, 30, or 50 % *w*/*v*) was dissolved in ethanol and heated up to 30 ± 1 °C in a water bath in a closed vessel. Afterwards, twice-distilled water was slowly added to the ethanolic solution up to a final 70:30 (*v*/*v*) ratio, under continuous stirring at 700 rpm by an IKA Eurostar digital (IKA Labortechnik Janke & Kunkel, Staufen, Germany). Mechanical stirring was performed for 30 min at room temperature in the dark. In the case of CoQ10 containing ETHO (ETHO-CoQ10), CoQ10 (1 mg/mL) was added to PC ethanol solution before the addition of water.

### 2.4. Characterization of Ethosomes

#### 2.4.1. Cryo-Transmission Electron Microscopy (Cryo-TEM)

Samples were vitrified as previously described [32]. Particularly a 2 µL sample droplet was put on a lacey carbon filmed copper grid (Science Services, Munich) for 30 s. Subsequently, a blotting paper has been employed to remove most of the liquid, resulting in a thin film stretched over the lace holes. The specimens were instantly shock frozen by rapid immersion into liquid ethane cooled to approximately 90 K by liquid nitrogen in a temperature-controlled freezing unit (Zeiss Cryobox, Carl Zeiss Microscopy GmbH, Jena, Germany). All the steps of sample preparation have been conducted at controlled temperature. After freezing the specimens, the remaining ethane has been removed by blotting paper. The vitrified specimen was transferred to a Zeiss/Leo EM922 Omega EFTEM (Zeiss Microscopy GmbH, Jena, Germany) transmission electron microscope using a cryoholder (CT3500, Gatan, Munich, Germany). Sample temperature was maintained below 100K during the examination. Specimens were examined with reduced doses of about 1000–2000 e/nm^2^ at 200 kV. Images have been recorded by a CCD digital camera (Ultrascan 1000, Gatan, Munich, Germany) and analyzed using a GMS 1.9 software (Gatan, Munich, Germany). 

#### 2.4.2. Photon Correlation Spectroscopy (PCS)

Vesicle size analysis has been conducted using a Zetasizer Nano S90 (Malvern Instr., Malvern, England) with a 5 mW helium neon laser and a wavelength output of 633 nm. Measurements were taken at 25 °C at a 90 ° angle and a run time of at least 180 s. Samples have been diluted with twice distilled water in a 1:10 *v:v* ratio. Data were analyzed using the “CONTIN” method [33]. Measurements were performed thrice from 1 to 90 days months from ETHO production.

#### 2.4.3. X-ray Diffraction

Small angle X-ray scattering (SAXS) experiments were performed at the bioSAXS beamline B21 (Diamond Light Source, Harwell, United Kingdom). CoQ10 loaded and unloaded ETHO were transferred into PCR tubes in an automated sample changer. The samples were then delivered into a temperature-controlled quartz capillary and exposed for 1s, acquiring 30 frames at 20 °C in order to check equilibrium conditions and to eventually monitor radiation damage. Data were collected using a Pilatus Dectris 2 M detector with a 3.9 m sample–detector distance and X-ray wavelength λ = 1.0 Å. The explored Q-range (being Q the modulus of the scattering vector, defined as 4π sin θ/λ, where 2θ is the scattering angle) extended from 0.0004 to 0.035 nm^−1^. 2D data were corrected for background, detector efficiency, and sample transmission were then radially averaged to derive I(Q) vs. Q curves [34].

### 2.5. CoQ10 Content of Ethosomes

The entrapment capacity (EC) of CoQ10 in ETHO has been determined after 1, 30, and 90 days from production of ETHO-CoQ10. Namely a 500 µL sample was loaded in a centrifugal filter (Microcon centrifugal filter unit YM-10 membrane, NMWCO 10 kDa, Sigma-Aldrich, St. Louis, MO, USA) and subjected to ultracentrifugation (Spectrafuge™ 24D Digital Microcentrifuge, Woodbridge, NJ, USA) at 8,000 rpm for 20 min. Hundred microliters of ETHO-CoQ10 in the supernatant have been diluted with ethanol (1:10, v/v) and maintained under magnetic stirring for 30 min [35]. After filtration of the obtained ethanol solution by nylon syringe filters (0.22 µm pores), the amount of CoQ10 has been quantified by RP-HPLC, as above reported. The EC was determined as

EC = CoQ10 /T_CoQ10_ × 100 (1)

where CoQ10 corresponds to the amount of drug measured by HPLC and T_CoQ10_ is the total amount of CoQ10 employed for ETHO production.

### 2.6. Cell Culture and Cytotoxicity Studies

Fibroblast cells (primary dermal fibroblasts isolated from skin biopsy of a healthy control subject) were grown in Dulbecco’s modified Eagle’s medium (DMEM) Low Glucose, supplemented with 10% FBS, 100 U/mL penicillin, 100 μg/mL streptomycin and 2 mM L-glutamine. Cells were incubated at 37 °C for 24 h in 95% air/5% CO_2_ until 80% confluence. Stock solutions containing CoQ10 (500 μM) were further diluted to reach the final concentrations, ranging from 1 to 41.3 μM. For LDH assay seeded cells were treated for 24 h with the different vehicles at various concentrations. Cytotoxicity was evaluated by spectrophotometric quantification of the LDH released in culture medium, using a commercial kit (Sigma-Aldrich, Merck, Darmstadt, Germany), as previously described [36,37]. For MTT assay seeded cells were exposed to the selected formulations for 24 h, after complete removal of the treatment, 50 μL of serum-free media and 50 μL of MTT (0.5 mg/mL) were added and incubated for 3 h. The MTT was reduced in living cells, forming insoluble purple formazan crystals that were then dissolved in 100 μL of DMSO at 37 °C for 15 min. After shaking, the solution absorbance, being proportional to the number of living cells, was measured at 590 nm, using 670 nm as a reference wavelength, thus converted into percentage of viability [38]. In trypan blue exclusion assay, seeded cells were treated for 24 h with the different vehicles at various concentrations. Afterwards, detached cells were diluted 1:1 with trypan blue (0.5% solution), then 10 μL of cell suspension were applied on a Bürker Chamber for counting the living cells with an inverted microscope. The results were reported in the number of viable cells and the percentage was calculated in relation to non-treated cells, used as control (100% viability).

### 2.7. Reconstitution of Human Epidermal Equivalents

Human epidermal equivalents (HEEs) were reconstituted according to the CITYCARE HEE protocol from Dijkhoff et al [39]. Briefly, neonatal cells have been selected for their lack of environmental exposure, namely cells from donor of Caucasian ethnicity were preferable to limit the production of melanin. Culture of primary keratinocytes was initiated in complete KGM-Gold medium (Lonza, Walkersville, MD, USA). Before reaching confluency, proliferating keratinocytes were harvested by trypsinization, frozen, and preserved in liquid nitrogen. Third-passage proliferating keratinocytes were used for the reconstruction of the epidermis. Cell suspensions containing approximately 1 million cells were diluted in complete KGM™ Gold medium in two 75 cm^2^ culture flask for 10 h and then in EpiLife™ (Gibco™, Life Technologies, Carlsbad, CA, USA) basal medium containing low calcium level (60 µM CaCl_2_) which was renewed after 2 days. When keratinocytes were subconfluent (usually on the fifth day after thawing), cells were harvested by trypsinization and centrifuged for 5 min. The pellet was resuspended in EpiLife™ submerged medium containing 1.5 mM CaCl_2_. Polycarbonate culture inserts (0.47 cm^2^ of diffusion area and 0.4 µm pore size) (Nunc™, ThermoFisher Scientific, Tustin, CA, USA) were placed in a 24-well plate containing 1.5 mL of pre-warmed submerged medium, using a specific carrier system (Nunc™, ThermoFisher Scientific, Tustin, CA, USA). Each insert received about 500 µL of keratinocyte suspension to reach a cell density of 375.000 cells/cm^2^. After three-day incubation at 37 °C in a humidified atmosphere containing 5% CO_2_, cells were exposed to the air–liquid interface by removal of the upper compartment medium. The 1.5 mL of submerged medium in the lower compartment were replaced with 1.5 mL EpiLife™ air-liquid interface medium containing 1.5 mM CaCl_2_, vitamin C, and keratinocyte growth factor, and renewed every 2–3 days for 14 days.

### 2.8. Transmission Electron Microscopy (TEM) Analyses

Fibroblasts treated with ETHO for 24 h and non-treated cells, used as control, were grown to confluent monolayer on 10 cm Petri dish. RHE tissues were topically treated with ETHO for 6 h and non-treated RHE were used as control. Fibroblasts and RHE tissues were fixed in 2.5% glutaraldehyde in cacodylate buffer for 3 h at 4 °C. Then fibroblasts and tissues were post-fixed in 1% osmium tetroxide for 2 h at 4 °C, dehydrated in a graded series of alcohol, embedded in Araldite resins, and polymerized in oven for 48 h at 60 °C. Ultrathin sections of 60 nm were cut with an ultramicrotome (Ultratome Reichert SuperNova Leica, Wien, Austria), stained with uranyl acetate and lead citrate and examined in a Philips CM100 transmission electron microscope [40].

### 2.9. Immunocytochemistry

Fibroblasts were grown on coverslips at a density of 1 × 105 cells/mL, after 24 h of treatment with plain ETHO and ETHO-CoQ10, cells were exposed to 50 μM H_2_O_2_ for 1 h. After H_2_O_2_ exposure, the medium was changed with fresh medium and the cells were analyzed at three different time points (i.e., directly post exposure, 2 h and 6 h post-exposure). Afterwards, cells were fixed in 4% paraformaldehyde for 10 min at room temperature (RT). Cells were then permeabilized for 5 min at RT with PBS containing 0.2% Triton X-100, then the coverslips were blocked in PBS containing 1% BSA at RT for 1 h. Cells were then incubated with primary antibody for 4HNE (AB5605; Millipore Corporation, Burlington, MA, USA) (1.200) in PBS containing 0.5% BSA at 4 °C overnight. After washing, coverslips were incubated with appropriate secondary antibody (1:100) for 1 h at RT. Nuclei were stained with 1 μg/mL DAPI (Sigma-Aldrich, Merck, Darmstadt, Germany) for 1 min. Coverslips were mounted onto glass slides using anti-fade mounting medium 1,4 diazabicyclooctane (DABCO) in glycerin and examined by the Leica light microscope equipped with epifluorescence at 40× magnification. Negative controls for the immunostaining experiments were performed by omitting primary antibody. Images were acquired and analyzed with Leica software [41].

### 2.10. Immunohistochemistry

RHE was topically pretreated with 50 µL of ETHO-CoQ10 for 24 h, then exposed to 200 μM H_2_O_2_ for 90 min and analyzed. Formalin-fixed paraffin-embedded (FFPE) sections were used to quantify 4-HNE expression in the RHE models with a thickness of 4 µm. FFPE sections were deparaffinized, rehydrated, and rinsed with water. An antigen retrieval step was performed by incubating the FFPE sections in 10 mM citrate buffer pH 6 at 90 °C for 20 min. All FFPE sections were firstly washed twice in phosphate buffer saline (PBS), then blocked for 1h in PBS containing 0.2% bovine serum albumin and 0.02% Triton X-100, further incubated overnight at 4 °C with 4-HNE antibody (AB5605, Merck Millipore, Burlington, MA, USA) (1:100). Tissue sections were washed in PBS containing 0.2% bovine serum albumin and 0.02% Triton X-100 before incubation with Donkey Anti-Goat IgG H&L (Alexa Fluor^®^ 488; ab150129, Abcam, Cambridge, UK) (1:200) for 2h. After several washes, sections were finally stained with a diamidino-2-phenylindole dye (DAPI, Tocris Biosciences, Bristol, UK) 300 mM solution for 5 min, then washed thrice in TBS-T, a mixture of tris-buffered saline (TBS) and polysorbate 20 (aka Tween 20). Coverslips were subsequently mounted in an aqueous mounting medium (R&D Systems, Minneapolis, MN, USA) and tissue sections observed under a fluorescence microscope (Nikon Microphot FXA microscope; Nikon Instruments, Amsterdam, Netherlands) equipped with a fluorescein isothiocyanate (FITC) filter. DAPI and FITC pictures were finally overlapped [42].

### 2.11. Statistical Analysis

Statistical differences of in vivo data have been calculated by repeated-measures analysis of variance (ANOVA) and the Bonferroni–Dunn post hoc pairwise comparison procedure. The software Prism 5.0, Graph Pad Software Inc. (La Jolla, CA, USA) has been employed. Probability values less than 0.05 have been considered significant in this study.

## 3. Results

### 3.1. Solubility Study

In order to design a nanotechnological formulation suitable for the delivery of the lipophilic CoQ10 (log P 19.4, [43]), its solubility has been investigated in different solvents, namely water, ethanol, methanol, dimethyl sulfoxide, and acetonitrile. The drug was insoluble in almost all the solvents, while a slight solubility has been observed in ethanol, namely 0.3 ± 0.2 mg/mL. Since the addition of PC (3%, *w*/*v*) to ethanol enabled to increase CoQ10 solubility up to 1 mg/mL, the inclusion of the drug in ETHO vesicles has appeared the most appropriate strategy.

### 3.2. Preparation and Characterization of Ethosomes

**Figure 1:**
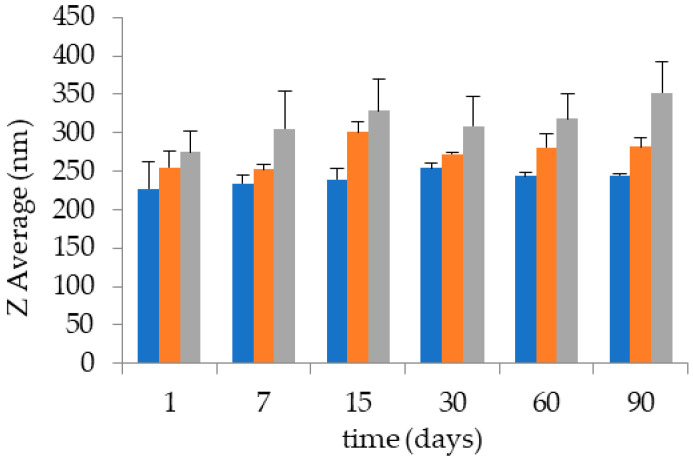
Effect of PC concentration on the stability of ETHO_0.6_ (orange), ETHO_0.9_ (blue), and ETHO_1.5_ (grey). Diameters were measured by PCS and expressed as Z average.

PC is a natural amphiphilic molecule widely employed for its emulsifying properties. In addition, PC can self-organize in bilayers in excess water, giving rise to supra-molecular structures, such as direct and inverted micelles; hexagonal, cubic, and lamellar phases; as well as stable vesicles. Thus, the addition of water to PC ethanol solution under stirring spontaneously led to ETHO, appearing as milky homogeneous dispersions [44]. Different PC concentrations have been considered in order to select the ETHO composition, namely a preformulation study has been conducted investigating the influence of PC 0.6, 0.9, and 1.5 % *w*/*w* on ETHO size, as reported in Table 1 and Figure 1.

It was found that both ETHO opalescence and mean diameter increased with the increasing of PC concentration, particularly ETHO mean diameters, measured by PCS, were comprised between 227 and 274 nm. Notably, ETHO size distribution was homogeneous, being characterized always by one peak, as indicated by the intensity distribution and by the low dispersity indexes (Table 2). ETHO size variation has been investigated by time, in order to gain information on their stability. After 3 months from ETHO preparation, ETHO size was almost stable, displaying an increase of 17, 27 and 78 nm (i.e., 7, 10, and 28%), respectively for PC 0.6, 0.9, and 1.5% *w*/*w*. Thus the lowest the PC concentration, the highest the size stability, as found by other authors [21].

Despite PC 0.6% enabled to better control ETHO size stability, since previous studies have demonstrated that phospholipid concentration in ETHO significantly affects drug entrapment efficiency [21,45,46], as a compromise, PC 0.9% *w*/*w* has been selected in order to adequately entrap CoQ10, while maintaining the vesicle size stability.

**Figure 2:**
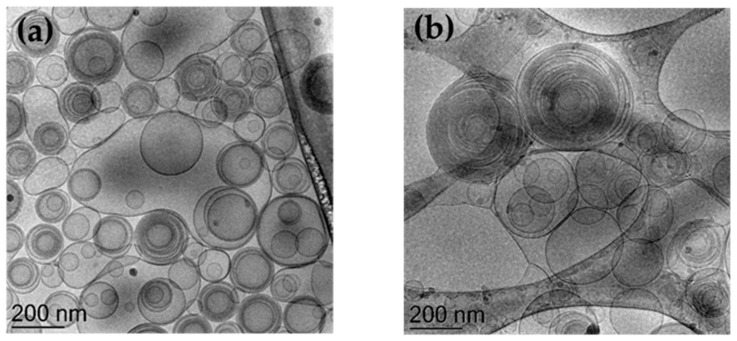
Cryo-transmission electron microscopy images (Cryo-TEM) of ETHO_0.9_ (**a**) and ETHO-CoQ10 (**b**). Bar corresponds to 200 nm.

Therefore, for ETHO-CoQ10 production the drug has been solubilized in PC 0.9% *w*/*w* ethanol solution, before water addition at 22 °C. This strategy, devoid of high energy input and heating, appeared particularly suitable for encapsulation of labile drugs such as CoQ10. The size analysis of ETHO-CoQ10 confirmed a unimodal size distribution also in the presence of the drug, with a 16 nm (6%) increase in the vesicle mean diameter, passing from 254 to 271 nm (Table 2). It is noteworthy that ETHO-CoQ10 mean diameter was almost unvaried at least for 3 months, undergoing merely a 22 nm (8%) increase. Cryo-TEM analyses have been conducted to gain information on ETHO morphology. In Figure 2 are reported images of ETHO_0.9_ (panel a) and ETHO-CoQ10 (panel b), clearly showing spherical vesicles. In both panels a mixed population of unilamellar, multilamellar, as well as multivesicular vesicles can be noted, particularly, a ‘fingerprint-like’ structure due to the presence of phospholipid bilayers, can be detected in both panels, as previously described by Touitou and Godin [47]. Notably, the presence of CoQ10 did not change the PC supramolecular organization of ETHO.

#### X-ray Diffraction

**Figure 3:**
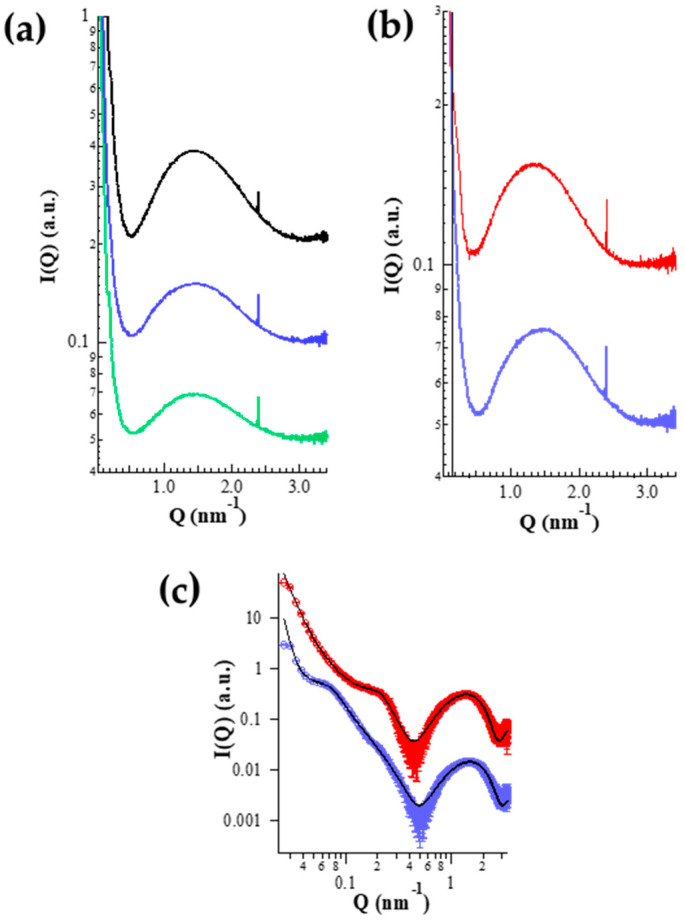
Small angle X-ray scattering (SAXS) profiles observed for the different ETHO samples at 25 °C. (**a**) ETHO_0.6_ (green), ETHO_0.9_ (blue), ETHO_1.5_ (black). (**b**) ETHO-CoQ10 (red), ETHO_0.9_ (blue). (**c**) Fitting analysis of the ETHO-CoQ10 (red) and ETHO_0.9_ (blue) curves by the new GENFIT model [50]. The continuous black lines are the best fit curves; the fitted Q-range corresponds to the length of the traced lines. In each frame, curves are scaled for clarity.

SAXS analyses have been conducted to shed light on the inner ETHO organization. Figure 3a reports the obtained scattering profiles for ETHO_0.6_, ETHO_0.9_ and ETHO_1.5_, showing a broad band (Q ≈ 1.5 nm^−1^) and two shoulders (Q ≈ 0.075 and 0.2 nm^−1^). The broad band, whose position roughly corresponds to a bilayer thickness of 4.2 nm, is indicative of the presence of PC bilayers [48,49], while the absence of distinct Bragg peaks suggests that the multilamellar vesicles are characterized by a large degree of disorder. Concerning the broad band, two points should be noticed (i) its position resulted independent of PC concentration, confirming its relationship with the bilayer electron density profile; (ii) its intensity was a linear function of the PC concentration, suggesting that the number of bilayers forming the vesicles increased with PC content. The SAXS curve of ETHO_0.9_ (also shown in Figure 3b) has been fitted by using a new GENFIT model [50] based on polydisperse spheres with core radius R_0_ and dispersion ξ_Ro_ (dispersion is described by a Schultz function [50]) surrounded by N bilayers. In this model, the core of the vesicle is filled with ethanol (constant electron density ρ_eth_) and the bilayers—symmetrically constituted by three shells with constant electron densities corresponding to the polar head (thickness R_1_ and electron density ρ_1_), the aliphatic chain (R_2_ and ρ_2_), and the terminal methyl group (R_3_ and ρ_3_) domains—are separated by water layers of thickness c and electron density ρ_0_. Moreover, the bilayer distribution is described by the paracrystal theory [50], with distortion parameter g_c_. The best fitting curve is reported in Figure 3c, while the fitting parameters are reported in Table 3. Notably, the number of bilayers, as well as the vesicle size, agree with the PCS and cryo-TEM results. In addition, the paracrystal distortion parameter reflects the ETHO disordered structure shown in Figure 2a.

SAXS profile of ETHO-CoQ10 (Figure 3b) was similar to that of unloaded ETHO (Figure 3a), except for a shift in the position of the maximum of the broad band, moving from 1.5 nm^−1^ (4.2 nm) to 1.38 nm^−1^ (4.6 nm) and for the disappearance of the first shoulder at Q = 0.075 nm^−1^. The change in the broad-band position suggests an increase of the lipid bilayer due to the CoQ10 presence, while the absence of distinct Bragg peaks confirms the disordered structure of ETHO vesicles. Model fitting results, reported in Figure 3c and Table 3, enabled to define at 0.4 nm the small enlargement of the PC bilayer, due to the dissolution of CoQ10 in the hydrocarbon lipidic region. In addition, model fitting evidenced a main supramolecular organization of multilamellar vesicles unaffected by the presence of CoQ10, as already evidenced by cryo-TEM (Figure 2b).

### 3.3. Entrapment Capacity of CoQ10 in ETHO

The EC of CoQ10 in ETHO was almost total, namely 98 ± 0.2% of the employed drug has been found within ETHO-CoQ10. This high EC value was expectable, indeed the preparation modalities prevented drug loss on mechanical devices, as well as thermal or light degradation. In addition many authors have demonstrated that the presence of ethanol and PC organized in multilamellar vesicles enables to increase drug loading with respect to other nanosystems [21,22,47]. As a matter of fact, it should be underlined that other authors have proposed the encapsulation of CoQ10 within nanosystems, such as solid lipid nanoparticles and liposomes. Nevertheless, ETHO were more effective, indeed EC values for CoQ10 reached at most 73% or 89% respectively in solid lipid nanoparticles and liposomes [16]. Therefore, the higher CoQ10 EC obtained by ETHO could be ascribed both to the supramolecular structure of ETHO and to the production modalities. 

CoQ10 stability has been studied evaluating EC values for 3 months in ETHO-CoQ10 stored in the light at 22 °C. It is noteworthy that, despite CoQ10 photo instability, EC was 90% and 60% after 30 and 90 days from production, thus higher with respect to similar studies conducted on nanostructured lipid carriers containing CoQ10 [19]. It can be hypothesized that in ETHO both phosphatidylcholine and ethanol organized in multilamellar vesicles contribute to create a strong interaction with CoQ10, controlling its degradation.

### 3.4. Cytotoxicity of ETHO Containing CoQ10

**Figure 4:**
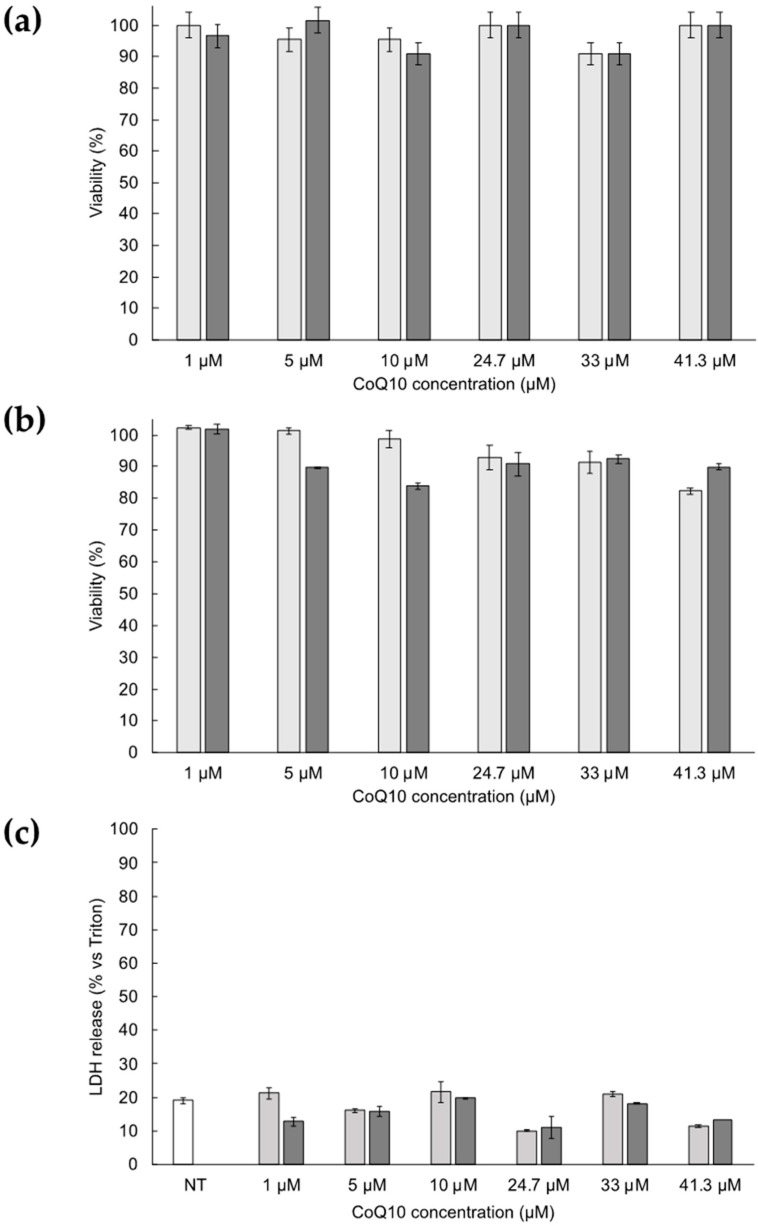
Cytotoxicity of ETHO_0.9_ (light grey) and ETHO-CoQ10 (dark grey) evaluated by trypan blue exclusion (**a**), MTT (**b**), and LDH (**c**) tests on primary dermal fibroblasts from healthy control subject after 24 h of treatment. Data are given as mean ± SD, representative of three independent experiments with at least three technical replicates each time.

The in vitro cytotoxicity of ETHO-CoQ10 has been assessed with different concentrations of ETHO_0.9_ and ETHO-CoQ10 (referring to loaded CoQ10) on primary dermal fibroblasts from healthy controls, using three different techniques: trypan blue exclusion, MTT, and LDH assays. The concentration range was selected in order to confirm the safeness and biocompatibility of ETHO constituents and of the loaded drug. Figure 4 displays the results of cytotoxicity obtained with the techniques above mentioned, comparing ETHO_0.9_ with ETHO-CoQ10. Both formulations display similar behavior, with no cytotoxicity up to the highest concentration tested. In detail, cell viability detected by trypan blue exclusion assay (Figure 4a) was higher than 90% for both ETHO_0.9_ and ETHO-CoQ10 for all concentrations. Moreover, MTT (Figure 4b) and LDH (Figure 4c) assays confirmed the absence of cytotoxicity, resulting in a cell viability of 82% and 89% in the case of MTT and LDH release of 11% and 13% respectively for ETHO_0.9_ and ETHO-CoQ10. Hence, the encapsulated CoQ10 did not induce toxic effects on cells at all concentrations tested. Based on these data, the highest CoQ10 concentration, (i.e., 41.3 µM) has been selected for further ex-vivo experiments.

### 3.5. ETHO Uptake in Fibroblasts Detected by TEM

**Figure 5:**
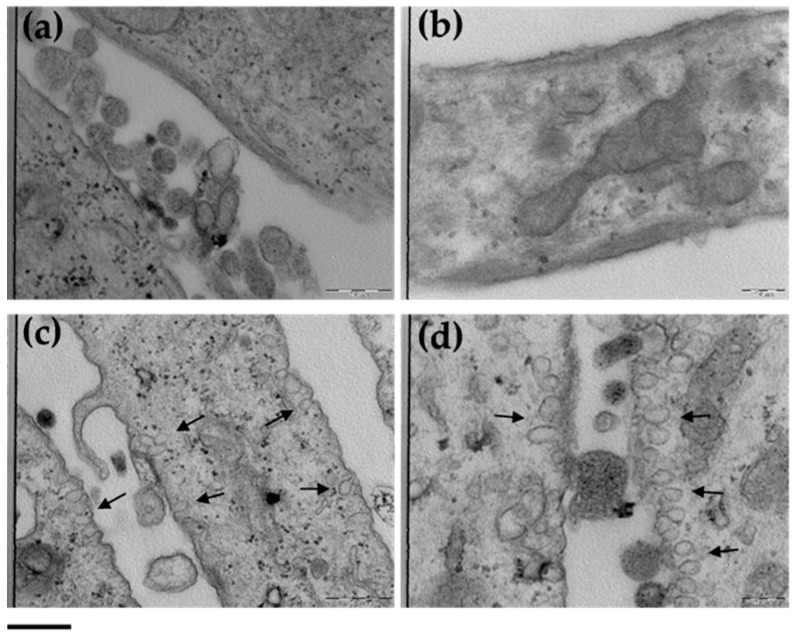
TEM images of control cells (**a**,**b**) and cells treated with ETHO_0.9_ (**c**,**d**). Images of panels (a) and (c) refer to 31.5K magnification; images of panels (b) and (d) refer to 50K magnification. Arrows indicate the presence of ETHO_0.9_. Bar corresponds to 500 nm.

In order to investigate the uptake of ETHO_0.9_ in treated fibroblasts, TEM analysis was performed, comparing the ultrastructural morphology with untreated cells (Figure 5).

Focusing on cell membrane section at the two experimental conditions and at different magnifications, the passage of ETHO_0.9_ is clearly evidenced in panels c and d. Moreover, the involvement of endocytosis pathway could be supposed for the presence of cell membrane invaginations and vesicles within the cytoplasm. Furthermore, comparing the images with those of untreated cells, it is noteworthy that the ultrastructural morphology of fibroblasts was not affected by the presence of ETHO_0.9_. These findings corroborate previous studies conducted by many authors, indicating that the malleable ETHO vesicles can penetrate cellular membrane, releasing the loaded molecule within cells [22,24,51,52,53,54].

Taking together the results of cytotoxicity and TEM, ETHO appeared as promising vesicle systems able to deliver the CoQ10 to the target cell, with the aim of enhancing the cellular antioxidant defense status.

### 3.6. Evaluation of 4HNE Protein Adducts Levels in Fibroblasts Treated with ETHO-CoQ10

**Figure 6:**
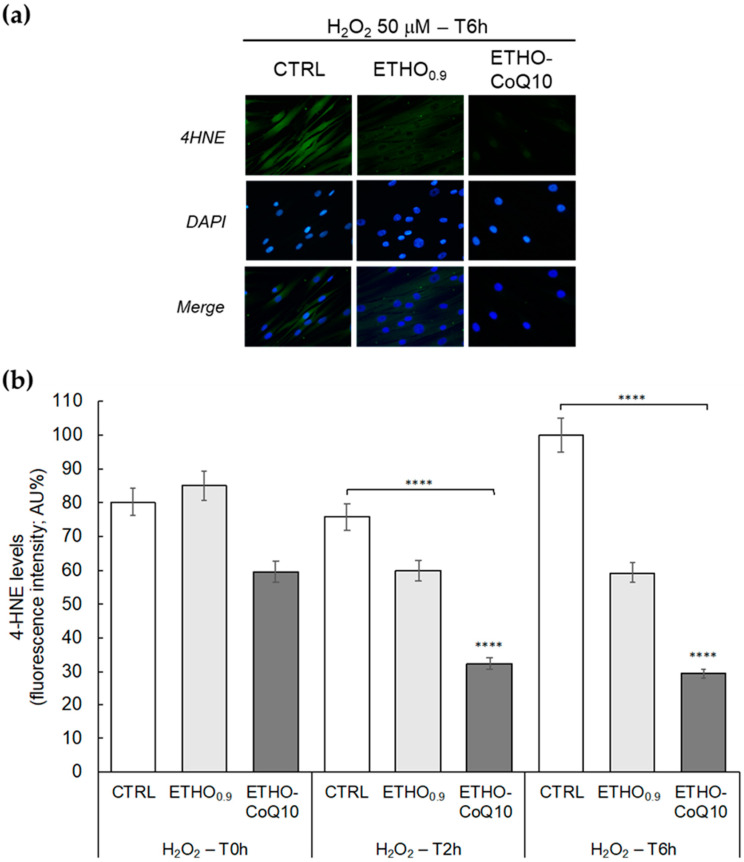
(**a**) Representative immunocytochemical images of 4HNE protein adducts in primary dermal fibroblasts without treatment (CTRL), treated with ETHO_0.9_ or ETHO-CoQ10, 6 h after exposure with 50 µM of H_2_O_2_ for 1 h. Images were taken at 40×. (**b**) Quantification of immunofluorescence staining for 4HNE immediately after H_2_O_2_ exposure (T0 h) or 2 (T2 h) and 6 h (T6 h) post-exposure. Data were normalized with respect to the CTRL sample treated for 6 h and expressed as arbitrary units ± SD. **** *p* ≤ 0.0001 vs. CTRL T0 h.

The ability of ETHO-CoQ10 in protecting cells against oxidative insults has been investigated by immunocytochemical analysis for 4HNE protein adducts on human health fibroblasts treated with H_2_O_2_, as an inducer of oxidative stress. 4HNE is an electrophilic α,β−unsaturated aldehyde, a reactive lipid mediator generated from oxidative stress-induced lipid peroxidation [55]. Briefly, untreated cells (CTRL), cells treated with ETHO_0.9_ and cells treated with ETHO-CoQ10 41.3 µM have been exposed to H_2_O_2_ 50 µM for 1 h. 4HNE protein adducts levels have been evaluated immediately after H_2_O_2_ exposure (T0 h), or 2 (T2h) and 6 (T6 h) h post-exposure. 4HNE protein adducts were analyzed by immunofluorescence (Figure 6a) and quantified with respect to untreated cells (Figure 6b). Comparing the results with those of cells not subjected to oxidative insult (data not shown), the exposure to H_2_O_2_ induced a significant increase in 4HNE protein adducts formation (green fluorescence), in CTRL and cells treated with ETHO_0.9_ cells (Figure 6a). On the other hand, when cells exposed to H_2_O_2_ were pre-treated with ETHO-CoQ10, the formulation was able to counteract the formation of 4HNE protein adducts, as indicated by the reduction in green fluorescence signal intensity (Figure 6a). As displayed in Figure 6b, after H_2_O_2_ exposure, the levels of 4HNE protein adducts in ETHO-CoQ10 treated cells were maintained around 30% even 6 h post-exposure, while the production of 4HNE adducts increased significantly over time in the untreated cells. This result agrees well with the TEM evidence, suggesting that ETHO-CoQ10—penetrated intact within fibroblasts—can exert antioxidative potential, protecting cells against oxidative stress [56]. 

### 3.7. ETHO Skin Uptake in Reconstituted Human Epidermis (RHE) Detected by TEM

**Figure 7:**
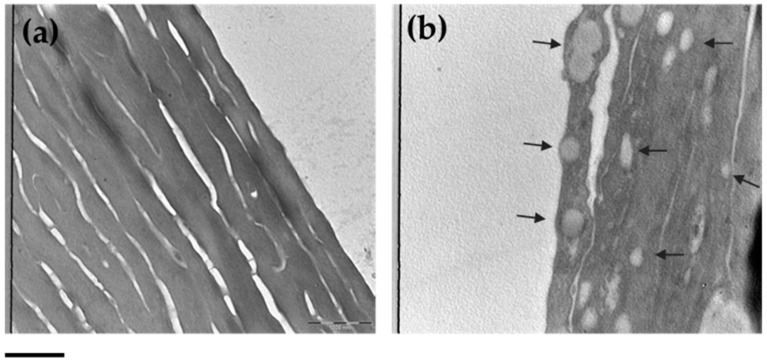
TEM images of control RHE (**a**) and RHE after 6 h of treatment with ETHO_0.9_ (**b**). Images refer to 20K magnification. Arrows indicate the presence of ETHO_0.9_. Bar corresponds to 1000 nm.

It is well-known that the complex structure of the skin acts a barrier and limits the passage of lipophilic molecules. Particularly, the outermost epidermal layer, the stratum corneum (SC), is the main hurdle for the substance passage to the deeper skin layers [57]. In this regard, RHE can represent a 3D model suitable for mimicking the human epidermis morphology and physiology, even after topical application of compounds [58]. Therefore, RHE was chosen to investigate the ability of ETHO in crossing the epidermal layers. Particularly, after topical treatment with ETHO_0.9_ for 6 h, RHE ultrastructural morphology has been detected by TEM (Figure 7). Figure 7b shows the presence of ETHO vesicles in the epidermal layers, clearly detectable as compared to untreated RHE taken as control (Figure 7a). The images confirmed that ETHO vesicles are able to pass through the SC cells of the RHE model.

### 3.8. Evaluation of 4HNE Protein Adducts Levels in RHE Treated with ETHO-CoQ10

**Figure 8:**
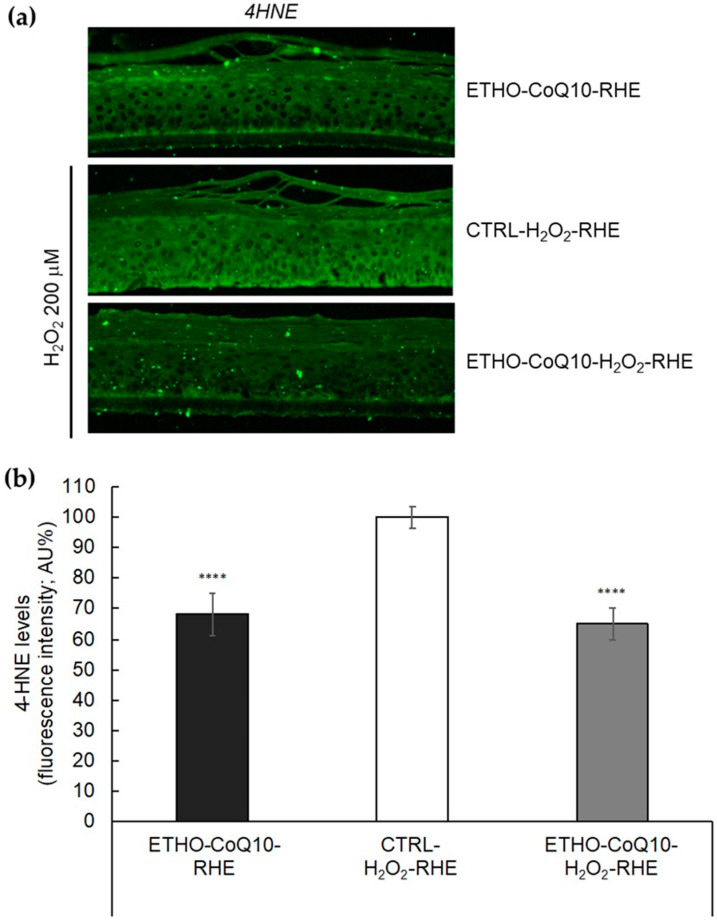
(**a**) Representative immunocytochemical images for 4HNE protein adducts in portions of RHE pretreated with ETHO-CoQ10 (ETHO-CoQ10-H_2_O_2_-RHE) and compared to control RHE treated with ETHO-CoQ10 not exposed to H_2_O_2_ (ETHO-CoQ10-RHE), or to RHE exposed to H_2_O_2_200 μM of H_2_O_2_ for 90 min (CTRL- H_2_O_2_-RHE). Images were taken at 40×. (**b**) Quantification of immunofluorescence staining for 4HNE after H_2_O_2_ exposure. Data were normalized with respect to CTRL-H_2_O_2_-RHE and expressed as arbitrary units ± SD. **** *p* ≤ 0.0001 vs. CTRL.

In order to investigate the ability of CoQ10 loaded ETHO in protecting skin tissue against oxidative stress, an immunohistochemical analysis for 4HNE protein adducts has been performed on RHE treated with ETHO-CoQ10 and exposed to H_2_O_2_. Particularly, the effect of H_2_O_2_ oxidative stress was detected in RHE pretreated with ETHO-CoQ10 (ETHO-CoQ10- H_2_O_2_-RHE) and compared to RHE treated with ETHO-CoQ10 and not exposed to H_2_O_2_ (ETHO-CoQ10-RHE), or to RHE exposed to H_2_O_2_ (CTRL- H_2_O_2_-RHE). 4HNE protein adducts levels have been analyzed by immunofluorescence (Figure 8a) and quantified with respect to untreated RHE (Figure 8b).

The exposure to H_2_O_2_ induced a significant increase in 4HNE protein adducts, as evidenced by the green fluorescence in CTRL-H_2_O_2_-RHE (Figure 8a). Notably, a 35% decrease in the fluorescence intensity of 4HNE protein adducts levels has been detected in the case of ETHO-CoQ10-H_2_O_2_-RHE, suggesting that ETHO-CoQ10 are able to protect skin tissue after exposure to oxidative stress (Figure 8b). It is noteworthy that the antioxidant effect of CoQ10 has been exerted not only on the superficial SC but also in the deeper layers, as detectable by the inhibition of the fluorescence throughout the whole RHE portion, down to the *stratum basale*. It can be supposed that after application, ETHO-CoQ10 first pass through the upper SC, then they reach the deeper layers, confirming the mechanism of skin penetration previously described for ETHO [51,54]. Indeed, the ability of ethanol as a penetration enhancer in disturbing the organization of SC lipids, promotes the diffusion of the soft malleable vesicles in the deeper skin layers, where their fusion allows the transdermal release of the drug [51,54].

## 4. Conclusions

The present investigation has demonstrated the suitability of ETHO as a transcutaneous delivery system for CoQ10. The preformulatory study has underlined the importance of size distribution analysis for selecting the composition of ETHO vesicles. SAXS and cryo-TEM evidenced the multilamellar structure of vesicles based on phosphatidylcholine and ethanol.

Notably, due to their peculiar supramolecular organization, ETHO were able to increase the EC of CoQ10 and to better control its stability with respect to other nanosystems previously investigated in other studies, confirming the tremendous potential of ETHO vesicles for solubilization and delivery of lipophilic drugs. The composition and preparation modalities of ETHO described in this study could be applied to entrap molecules not only with antioxidant properties in order to improve their absorption and to delay their degradation.

Ex-vivo studies have corroborated the hypothesis of using ETHO as nanosystems for CoQ10 delivery through the skin. Indeed, TEM analyses confirmed the ETHO fibroblasts uptake, as well as their passage through the more complex model RHE. Furthermore, the decrease of fluorescence signal intensity down to the *stratum basale* corroborates the efficiency of ETHO as transdermal delivery systems, resulting as a promising vehicle for CoQ10 encapsulation. Remarkably, RHE can be considered reliable models to investigate the effect of topical formulation on skin; nevertheless, further in vivo studies will be required to evaluate not only the comparable antioxidant delivery against other carrier systems, but also the prolonged antioxidant effect of ETHO-CoQ10.

## Figures and Tables

**Table 1:** Composition of ethosomes

Ethosome	PC ^1^	Ethanol % *w*/*w*	CoQ10 % *w*/*w*	Water % *w*/*w*
ETHO_0.6_	0.6	29.4	n.p.	70.0
ETHO_0.9_	0.9	29.1	n.p.	70.0
ETHO_1.5_	1.5	28.5	n.p.	70.0
ETHO-CoQ10	0.9	29.0	0.1	70.0

^1^ soy phosphatidylcholine; n.p. not present.

**Table 2:** Dimensional distribution parameters of ethosomes, as determined by PCS.

Ethosome	Days	Z Average ± s.d. (nm)	Typical Intensity Distribution		Dispersity Index ± s.d.
			nm	Area %	
ETHO_0.6_	0	227.45 ± 34.10	237.55	100	0.12 ± 0.02
	90	244.60 ± 5.30	242.11	100	0.14 ± 0.03
ETHO_0.9_	0	254.73 ± 21.0	244.68	100	0.10 ± 0.02
	90	282.04 ± 10.01	275.34	100	0.12 ± 0.03
ETHO_1.5_	0	274.60 ± 32.10	266.51	100	0.13 ± 0.02
	90	352.15 ± 22.01	327.53	100	0.22 ± 0.03
ETHO-CoQ10	0	271.20 ± 5.56	277.30	100	0.14 ± 0.01
	90	293.05 ± 17.18	278.42	100	0.16 ± 0.03

s.d.: standard deviation.

**Table 3:** Fitting parameters for ethosomes, as determined by SAXS model analysis

Ethosome	*R*_1_ (Å)	*R*_2_ (Å)	*R*_3_ (Å)	ρ_1_ e/Å^3^	ρ_2_ e/Å^3^	ρ_3_ e/Å^3^	*N*	c (Å)	g_c_	*Ro* (Å)	ξ_Ro_	*ρ*_et_ e/Å^3^	*d*_lip_ (Å)	*R_tot_* (Å)
ETHO_0.9_	5.2	10.3	2.3	0.43	0.31	0.29	3 ± 2	560	0.33	133	0.33	0.29	35.4	270
ETHO-CoQ10	6.9	11.9	1.2	0.42	0.30	0.29	9 ± 1	115	0.40	199	0.40	0.29	39.8	296

SAXS: Small angle X-ray scattering; *R*_1_*:* thickness of the polar head shell; *R*_2_: thickness of the aliphatic chain shell; *R*_3_: thickness of the terminal methyl group shell; *ρ*_1_: electron density of the polar head shell; *ρ*_2_: electron density of the aliphatic chain shell; *ρ*_3_: electron density of the terminal methyl group shell; *N*: number of stacked bilayers; *c*: water layer thickness; *g_c_*: bilayer paracrystal distortion parameter; *Ro*: core radius of the spherical vesicle; ξ_Ro_: Schultz dispersion parameter; *ρ_et_*: ethanol electron density; *d*_lip_, total thickness of the lipid bilayer; *R_tot_* vesicle total radius. Errors in *R*_i_ are ± 0.1 Å; errors in c and in g_c_ are in the order of 10%; errors in *R*_0_ are in the order of 5%; errors in *R*_tot_ are in the order of 10%.
